# From Safety Evaluation to Influencing Factors Analysis: A Comprehensive Investigation on Ocular Irritation of Baby Bath Products

**DOI:** 10.3390/toxics13110948

**Published:** 2025-11-03

**Authors:** Qidi He, Yurong Zhong, Peining Li, Yanhua Guo, Chengkai Mei, Dongmei Xu, Erping Yan, Shaofeng Xi, Guoshan He, Jianhua Tan

**Affiliations:** 1 Guangzhou Quality Testing and Inspection Institute, National Quality Supervision and Testing Center for Cosmetics (Guangzhou), Guangzhou 510000, China; anchorgreen@foxmail.com (Q.H.); lpn1117@163.com (P.L.); guoyanhuasysu@foxmail.com (Y.G.); vickiemay@163.com (C.M.); 13922262758@163.com (S.X.); 2 Collaborative Innovation Center for NQI-Quality Safety of Guangzhou, Guangzhou 510000, China; 3 Kangaroo Mommy Research Centre, Guangdong Kangaroo Mommy Biotechnology Co., Ltd., Guangzhou 510000, China; yulingqingheng@163.com (Y.Z.); v1126942960@163.com (D.X.); jsyan6944@icloud.com (E.Y.)

**Keywords:** baby bath products, ocular irritation, surfactants, typical usage concentration, sodium laureth sulfate, combination

## Abstract

Given the underdevelopment and sensitivity of babies’ eyes, frequently used baby bath products have garnered attention for their ocular irritation safety. The selection and application of raw materials are key factors. We tested 39 commercial baby bath products for ocular irritation safety and proposed a novel evaluation method using the “Risk Attention Index” to analyze formulation risk factors. The ocular irritation potential of 39 commonly used raw materials under typical usage conditions was investigated via animal and cell models. Results showed over 20% of commercial products had safety risks. Surfactants like cocamidopropyl betaine (CAPB), sodium laureth sulfate (SLES), and sodium lauroamphoacetate (SLA) had the highest “Risk Attention Index”, requiring special attention. Some fragrance components were also potential irritants. Combining SLES with panthenol, maltooligosyl glucoside/hydrogenated starch hydrolysate, and Tween^®^-28 could significantly reduce its irritation in an animal model. The same ingredients from different sources led to inconsistent irritation results. This suggests that manufacturing processes of raw materials, in addition to their chemical properties, concentration, and combination methods, all influence the ocular irritation potential. This study provides key scientific evidence for assessing ocular irritation and developing low-irritation formulations in baby bath products, thereby enhancing product safety.

## 1. Introduction

During routine cleansing procedures, baby bath products are highly prone to coming into contact with infants’ eyes. However, infants and young children have underdeveloped lacrimal glands and blink less frequently, which means they cannot secrete sufficient tears to protect their eyes [1]. Therefore, the safety requirements for baby bath products are stringent on a global scale. Chinese regulations stipulate that the results of the acute eye irritation test for children’s cosmetics must be non-irritating or slightly irritating, and only non-irritating products can be labeled as “tear-free formula.” The U.S. regulations explicitly restrict the ingredients of baby cosmetics and require safety assessments. Although Europe, Japan, and South Korea do not have explicit formula guidelines, they do impose restrictions on certain ingredients and require warning labels. The common thread across these regions is a universally stricter approach to children’s products compared to adult cosmetics. This shared principle manifests in stringent restrictions on harmful ingredients, requirements for comprehensive safety assessments, and an emphasis on strengthened manufacturer responsibility. The formulation design of children’s cosmetics should prioritize safety, using simple formulas and reducing the use of ingredients that are prone to cause allergies or have strong irritancy [2]. Surfactants, preservatives, and fragrances are common ingredients in baby bath products. Among them, surfactants, as key functional ingredients in cleansing products, not only determine the cleaning power of the product but also directly affect its safety. The selection and use of surfactants are, therefore, crucial [3].

Surfactants are multifunctional amphiphilic molecules that are widely used in cosmetics [4]. Based on the characteristics of hydrophilic and hydrophobic groups, they can be classified into four categories: cationic, anionic, amphoteric, and nonionic. Anionic surfactants have strong cleaning power and are commonly used as the main surfactants in cleansing products [3]; amphoteric and nonionic surfactants have lower irritation and are often used as co-surfactants; cationic surfactants are mainly used in disinfectant products [5]. Strongly irritating surfactants can disrupt cell membranes, cause inflammation, and lead to adverse reactions such as eye discomfort, skin irritation, and allergies [6].

The Draize test in rabbits is a classic method for evaluating the eye irritation of cosmetic ingredients and products [7,8]. It assesses the degree of eye irritation by evaluating the damage to the cornea, conjunctiva, and iris of the rabbit’s eye. Currently, global regulatory frameworks and industry are undergoing a paradigm shift aligned with the “3Rs” principles (Replacement, Reduction, Refinement), making the adoption of alternative in vitro methods an irreversible trend. Presently, several in vitro methods have been adopted by the OECD and are widely applied, including cell models (such as rabbit corneal epithelial cells (SIRC cells) [9,10] (OECD TG 491), alternative models (such as the hen’s egg test-chorioallantoic membrane (HET-CAM) [11,12]), reconstructed cornea models (EpiOcular™ and SkinEthic™) [13,14,15] (OECD TG 492), ex vivo organ models (such as bovine corneal opacity and permeability (BCOP) test [16] (OECD TG 437), ex vivo rabbit eye [17,18,19], ex vivo chicken eye [20,21], etc.) and integrated assessment testing approach (IATA) [22,23]. The European Centre for Toxicology and Ecotoxicology of Chemicals (ECETOC) published the first eye irritation reference chemical database in 1992 [24], which was expanded to 132 chemicals (149 in vivo studies) in 1998 [25]. In 2017, Cosmetics Europe and the European Commission compiled the external data from institutions such as ECETOC into the Draize Eye Test Reference Database (DRD), which includes 681 independent in vivo studies and covers the eye irritation safety classification information of 634 chemicals [26]. Luechtefeld et al. analyzed the REACH (Registration, Evaluation, Authorisation and Restriction of Chemicals) database made public by the European Chemicals Agency (ECHA), which contains 9782 Draize tests involving 3420 substances [27].

The experimental data in existing eye irritation databases are mostly tested on the undiluted solutions of cosmetic ingredients or at relatively high concentrations, without fully simulating the actual state of the ingredients in formulations, including concentration, pH, and combination with other ingredients. This limits the reference value of the test results in the development and safety evaluation of cosmetic product formulations. Moreover, with the continuous expansion of the cosmetic ingredients library, the components in the original database cannot comprehensively cover the existing ingredients, resulting in data gaps in the safety assessment of cosmetics and their ingredients.

To address the limitations of existing databases and enhance the safety of baby bath products, this study collected commercial baby bath products and core ingredients commonly used in formulations. The irritation of products and ingredients was evaluated through the Draize test in rabbits and cell models. Ingredients test samples were prepared by simulating typical usage concentrations and pH values. A new evaluation method based on “Eye Irritation Score” and “Risk Attention Index” was proposed to identify the irritation risks of ingredients in formulations. Additionally, strategies for effectively reducing eye irritation were explored through multi-ingredient combination studies. A comprehensive database was constructed to record key information such as the source of ingredients, chemical structure, formulation concentration, solution pH, and combination ratios. This database is designed to offer a robust scientific foundation for evaluating the ocular irritation potential of typical ingredients in baby bath products. It also aims to facilitate the optimization of formulations and the development of safer products.

## 2. Materials and Methods

### 2.1. Test Samples

Thirty-nine baby bath products and Thirty-nine commonly used ingredients were purchased from mainstream market brands (manufactured between 2021 and 2022).

### 2.2. Instruments and Reagents

An electronic balance (ACS-30) was purchased from Guangzhou Zhongxing, Guangzhou, China. The pH meter (ZD-2) was purchased from Inesa Scientific Instrument Co., Ltd., Shanghai, China. Handheld slit lamp (YZ3) was obtained from Suzhou 66 Vision, Suzhou, China. A low-speed centrifuge (SC-3612) was acquired from Zhongke Zhongjia, Hefei, China. A biological safety cabinet (model 2-B2) was purchased from Nuaire, Plymouth, USA. CO_2_ incubator (2406-2) was obtained from SHELLAB, Torrance, USA. An inverted phase-contrast microscope (AxioVert A1) was purchased from ZEISS, Oberkochen, Germany. Fully automatic cell counter (Scepter 2.0) and pure water system (Milli-Q) were obtained from Millipore, Dornstetten, Germany. Multifunctional microplate reader (VARIOSKAN LUX) was purchased from Thermo Fisher Scientific Inc., Waltham, MA, USA.

Cell culture medium, fetal bovine serum, and tumor necrosis factor-α (TNF-α) were purchased from Thermo Fisher Scientific Inc., Waltham, MA, USA. CCK-8 reagent (BMU106-CN) was obtained from Abbkine Inc., Wuhan, China. Puromycin and penicillin-streptomycin were purchased from Beyotime Biotech Inc., Shanghai, China. Pure water for experiments (18.2 MΩ·cm) was prepared using the Milli-Q pure water system, MilliporeSigma, St. Louis, MO, USA. The SIRC cell line was purchased from the American Type Culture Collection (ATCC), Manassas, VA, USA.

### 2.3. Animals

New Zealand white rabbits of the conventional grade were purchased from the Guangdong Provincial Medical Laboratory Animal Center (Sanshui Base, Foshan, China), with the production license number: SCXK (Yue) 2019-0035. After acquisition, the animals were individually housed in the conventional environment animal room of this institute [Experimental Animal Use License Number: SYXK (Yue) 2018-0137], with the temperature maintained at 18 °C to 26 °C and relative humidity at 40% to 70%. The feed was provided by Beijing Keao Xieli Feed Co., Ltd. (Beijing, China). [Production License Number: SCXK (Jin) 2020-0004]. The animal experiments were approved by the Animal Welfare and Ethics Committee of the Guangzhou Quality Supervision and Testing Research Institute (IACUC2021-12-01 and IACUC2022-12-02).

### 2.4. Draize Test

The eye irritation of products and ingredients was evaluated using the modified Draize test method [12,28] based on the OECD Guideline 405. The details were shown in the Supplementary Materials. Briefly, three conventional-grade New Zealand rabbits were selected for the acute eye irritation test of each test substance. The study was conducted in the conventional-grade animal room rabbit laboratory. The eye irritation response was classified into five grades: non-irritating, slightly irritating, mildly irritating, irritating, and corrosive, based on the mean of the irritation response scores and the recovery time. The scoring criteria are shown in Table S1.

### 2.5. Cell Culture

The SIRC cells were cultured in high-glucose minimum essential medium (MEM) containing 10% fetal bovine serum (with 2 mmol/L glutamine and appropriate antibiotics (final concentrations of 50–100 IU/mL penicillin and 50–100 μg/mL streptomycin)), under conditions of 37 °C and 5% CO_2_.

### 2.6. Short-Time Exposure Test of SIRC Cells

The irritation of surfactants and their combinations was evaluated according to the short-time exposure (STE) in vitro test method of SIRC cells [29]. The cell viability was tested using the CCK-8 staining method: after exposure for 5 min with the test sample, the cells were washed 2–3 times with 200 μL of phosphate-buffered saline. Then, 100 μL of culture medium and 10 μL of enhanced CCK-8 solution were added to each well, followed by incubation in a 37 °C 5% CO_2_ incubator for 40 min. Subsequently, the absorbance at 450 nm (OD_450_) was measured using a multifunctional microplate reader. The cell viability was calculated based on the OD value, with six replicates for each test concentration. The formula for calculating cell viability (1) is as follows:

(1) $$\mathrm{cell}\;\mathrm{viability}(\%)=100\times \frac{{\mathrm{OD}}_{450\text{sample}}-{\mathrm{OD}}_{450\text{blank}}}{{\mathrm{OD}}_{450\text{control}}-{\mathrm{OD}}_{450\text{blank}}}$$

### 2.7. pH Measurement

The pH meter was calibrated using standard buffer solutions (pH 4.00, 6.86, and 9.18) through a multi-point calibration procedure. After calibration, the electrode of the pH meter was immersed in the solution to be measured, ensuring that the electrode was clean and fully submerged. Once the reading on the pH meter stabilized, the displayed value was recorded as the pH of the solution.

### 2.8. Data Analysis

Data analysis was performed using SPSS 27.0 software (IBM Corp., Armonk, NY, USA), with the significance level α set at 0.05.

## 3. Results

### 3.1. Eye Irritation Test Results of Commercial Baby Bath Products

**Figure 1:**
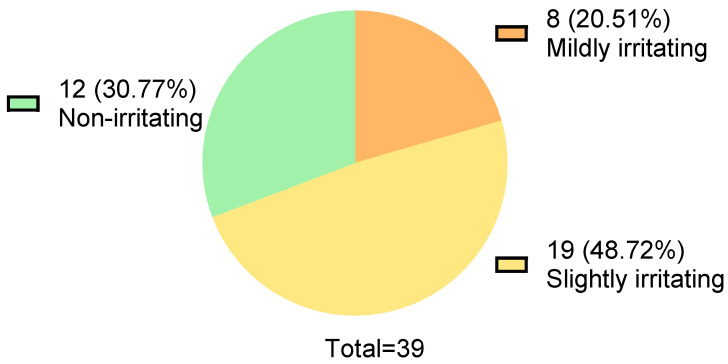
Draize test results of thirty-nine commercial baby bath products.

The Draize test results of 39 baby bath products (manufactured between 2021 and 2022) are shown in Figure 1. Twelve products were non-irritating, 19 products were slightly irritating, and 8 products were mildly irritating, accounting for 30.77%, 48.72%, and 20.51%, respectively (Figure 1). The formulations of the 39 products were collected from the official websites. The usage frequency of the top five ingredients in each formulation was counted, as well as the number of times for each ingredient appeared in non-irritating, slightly irritating, and mildly irritating formulations. The “Eye Irritation Score” and “Risk Attention Index” were calculated.

The formula for calculating the Eye Irritation Score (2) is:

Eye Irritation Score = [2 × Number of usage (Mildly irritating) + 1 × Number of usage (Slightly irritating) + 0 × Number of usage (non-irritating)]/Total number of usage.                   (2)

The formula for calculating the Risk Attention Index (3) is:

Risk Attention Index = Eye Irritation Score × Usage Rate × 100.(3)

Table 1 shows the top ten surfactants in terms of usage rate and their corresponding statistical data. Cocamidopropyl betaine (CAPB) is the most frequently used surfactant, appearing in 18 of the 39 formulations with a usage rate of 46.15%. It appeared 4 times in mildly irritating formulations, 13 times in slightly irritating formulations, and 1 time in non-irritating formulations, with an Eye Irritation Score of 1.17 and a Risk Attention Index of 54. PEG-120 methyl glucose dioleate ranked first in Eye Irritation Score (1.33), but due to its low usage rate (7.69%), the Risk Attention Index was only 10. The second and third highest Risk Attention Index values were obtained by sodium laureth sulfate (SLES) (26) and Sodium Lauroamphoacetate (SLA) (23), respectively, which also ranked third and fourth in Eye Irritation Score. PEG-80 sorbitan laurate (Tween^®^-28) had a high usage frequency (30.77%), but its Eye Irritation Score was only 0.67, resulting in a lower Risk Attention Index (21).

### 3.2. Eye Irritation Test Results of Common Ingredients in Formulations Under Typical Usage Conditions

Subsequently, we collected 39 commonly used raw materials in commercial baby bath products, including 31 surfactants, 3 preservatives, 4 fragrances, and 1 humectant. Samples were prepared based on typical usage concentrations and pH values in actual formulations, and their eye irritation was assessed using the Draize test. The results are shown in Table 2.

Anionic surfactants were tested 18 times, involving 14 different structures, with 3 instances of mild irritation, 6 instances of slight irritation, and 9 instances of non-irritation. Most of the 7 amino acid salt anionic surfactants exhibited non-irritating or slightly irritating properties. Sodium lauroyl sarcosinate (5%) was found to be mildly irritating. Sulfonate and carboxylate anionic surfactants exhibited weaker irritation, and within the tested concentration range of 1.44–3%, no irritation was observed. Only laureth-5 carboxylic acid and sodium taurine cocoyl methyltaurate showed slight irritation at higher concentrations (5%). Polyether sulfate anionic surfactants exhibited stronger overall irritation. Both ammonium laureth sulfate (5%) and sodium trideceth sulfate (3.25%) were mildly irritating, while sodium laureth sulfate (5%) was slightly irritating.

Amphoteric surfactants were tested 22 times (involving 9 different structures), with 7 instances of mild irritation, 5 instances of slight irritation, and 10 instances of non-irritation. The 7 instances of mild irritation occurred in tests of CAPB (test concentration 2%), lauryl hydroxysultaine (test concentration 2.4%, from 2 different sources), lauramidopropyl hydroxysultaine (test concentration 3.5%, from 3 different sources), and SLA (test concentration 5%). In the tests of CAPB and SLA, we found that the irritation of the same ingredient from different sources also varied, with results distributed across non-irritating, slightly irritating, and mildly irritating categories.

Nonionic surfactants were tested 7 times, involving 7 different structures. Only decyl glucoside (5%) exhibited slight irritation, while the remaining 6 surfactants showed no irritation under the tested conditions. The cationic surfactant polyquaternium-10 showed no irritation at a lower concentration (0.3%).

In addition, the humectant dipropylene glycol (test concentration 0.02%) and the three preservatives (test concentration 0.5–1.5%) showed no eye irritation in the tests. Phenoxyethanol at a test concentration of 1% did not elicit an irritation response, regardless of whether it was rinsed or not. Fragrances are typically mixtures of multiple components. The four fragrances tested in this study had varying results: Fragrance 369 (0.2%) was mildly irritating; orange blossom fragrance (0.1%) showed slight irritation; whereas red mandarin essential oil (0.1%) and Fragrance 440095 (0.2%) showed no irritation under the tested conditions.

### 3.3. Draize Test Results of Sodium Laureth Sulfate (SLES) Combinations

The combination of surfactants is a common strategy in cleansing products. It can not only enhance the synergistic effect and improve the dispersibility of the products, but also reduce irritation and the amount of surfactants used. In the combination system, the interaction between surfactants and active ingredients of different structures or types determines the performance and efficacy of the entire system. This study employed a two-component combination system to investigate the effects of different components on the eye irritation of 5% SLES.

Table 3 lists the combinations of SLES with other ingredients, their concentrations, pH values, and the results of the irritation tests using animal models. The pH values of most combinations were between 6.0 and 7.0, while the pH range for combinations with Tween^®^-28 and disodium cocoyl glutamate (DCG) was between 5.5 and 6.0.

The experimental results showed that different combinations had a significant impact on eye irritation. The irritation level of SLES (5%) combined with panthenol (1%), maltotriose glucoside/hydrolyzed starch hydrolysate (MG-60) (1.48%), and Tween^®^-28 (3.5%) was reduced from slightly irritating to non-irritating. The irritation level of SLES (5%) combined with polyquaternium-51 (PQ-51) (0.007%) remained slightly irritating. The irritation level of SLES (5%) combined with DCG (3%) increased from slightly irritating to mildly irritating.

### 3.4. Short-Term Exposure Eye Irritation Test Results of SLES Combinations Using the SIRC Cell Model

**Figure 2:**
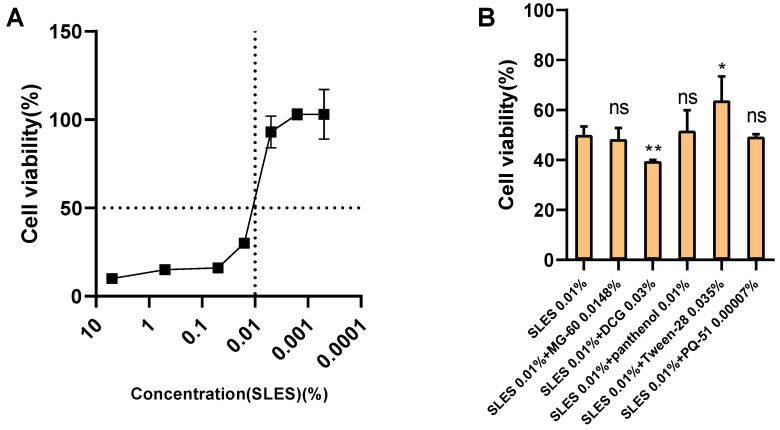
The effect of SLES combined with various substances on the viability of SIRC cells. (**A**) The cell viability of SIRC cells treated with SLES at various concentrations (0.0001–10%), (**B**) The cell viability of SIRC cells treated with 0.01% SLES and 0.01% SLES combined with different active ingredients. The cell viability was presented as mean ± standard deviation, *n* = 6. ns: no significant difference compared with the cell viability of the SLES 0.01% treatment group, ns: no significance, compared with the cell viability of the SLES 0.01% treatment group, *: *p* < 0.05, compared with the cell viability of the SLES 0.01% treatment group, **: *p* < 0.01, compared with the cell viability of the SLES 0.01% treatment group.

To further explore the reason why SLES combinations reduce irritation, we tested various combinations using the SIRC cell model. Given the differences in tolerance to surfactant toxicity between cells and animals, we first determined the appropriate experimental concentration of SLES through cytotoxicity testing. The results of the effect of SLES concentration on SIRC cell viability are shown in Figure 2A. Based on cell viability, we selected 0.01% SLES (with a cell viability of approximately 50%) as the main surfactant for the combinations. Subsequently, the combinations used in animal experiments were diluted 100 times, mixed with 0.01% SLES, and the pH was adjusted to 5.5–6.5 for the STE test in the SIRC model.

The results showed (Figure 2B) that compared with the 0.01% SLES treatment group, combinations of SLES with PQ-51 (0.00007%), panthenol (0.01%), and MG-60 (0.0148%) did not significantly affect the viability of SIRC cells. In contrast, the combination of SLES with DCG (0.03%) resulted in a significantly lower cell viability (39% ± 0.5%) than the 0.01% SLES treatment group (50% ± 5.7%). Moreover, the combination of SLES with Tween 28 (0.035%) significantly increased cell viability to 64% ± 8.6%.

## 4. Discussion

### 4.1. Eye Irritation and Formulation Analysis of 39 Commercial Baby Bath Products

Children’s skin and eyes differ significantly from adults physiologically. Their eyes are underdeveloped, with weaker defenses and more delicate structures, making them highly sensitive to external stimuli and prone to injury. Thus, evaluating eye irritation in children’s cosmetics is crucial.

The reliability of extrapolating acute eye irritation test results from animals to humans is very limited. White rabbits are generally more sensitive to irritant or corrosive substances than humans. However, the rabbit eye is a complex physiological system, comprising the cornea, conjunctiva, iris, as well as tear secretion and inflammatory responses. The rabbit eye irritation test can observe the integrated response of interactions among these tissues, which cannot be fully simulated by any single in vitro model at present. The Draize test has been in use for decades, accumulating a vast amount of historical data. Regulatory authorities and enterprises have a relatively mature understanding of the correlation between these data and potential human eye reactions, making it the “gold standard” for risk assessment for a long time.

Under Chinese regulations, children’s cosmetics must be slightly irritating or less. If a product claims to be tear-free, it must be non-irritating. However, tests on 39 children’s cosmetics produced in 2021–2022 showed that over 20% were mildly irritating, nearly half were slightly irritating, and only 30% met the non-irritating standard. This highlights the need for better formulation design in commercial children’s cosmetics (Figure 1).

We investigated the formulations of these products through the official websites. Although the published formulations did not explicitly list the specific concentrations of each ingredient, according to the requirements of Chinese regulations, the formulation submitted during the registration and filing of cosmetics must be sorted by the concentration of ingredients from highest to lowest. Therefore, we can infer the priority of each ingredient in the formulation based on the order of arrangement. On this basis, we proposed a new data processing method to more intuitively evaluate the irritation risk of ingredients in formulations through two indicators: Eye Irritation Score and Risk Attention Index. In the formula for calculating the Eye Irritation Score, we assigned scores of 2, 1, and 0 to mildly irritating, slightly irritating, and non-irritating formulations, respectively. The higher the Eye Irritation Score, the higher the proportion of the ingredient appearing in strongly irritating formulations, suggesting that the ingredient itself is more irritating. The Risk Attention Index is the product of the Eye Irritation Score and the usage rate. The higher the usage rate and the stronger the irritation of the ingredient, the higher the Risk Attention Index, and the more attention it requires in formulation applications.

Unlike conventional toxicological methods that test individual raw materials for irritation or standard protocols that merely conduct “black-box” testing of overall formulations, our approach is a “Formulation Structure Correlation Analysis.” It utilizes the ingredient ranking (concentration priority) and irritation results of tested formulations to infer the risk level of raw materials through statistical associations, thereby ingeniously incorporating the interactions within the overall formulation into the assessment.

The formulation analysis results (Table 1) showed that among the ingredients used in the 39 products, CAPB was the most frequently used surfactant. CAPB is an amphoteric surfactant with a hydrophilic betaine group (a positively charged quaternary ammonium cation group) and a lipophilic group derived from the long-chain fatty acids of coconut oil. CAPB was generally considered to be mild because the balance of positive and negative charges helps reduce potential damage to the skin and eyes [30]. However, statistical analysis showed that 4 of the 8 mildly irritating products contained cocamidopropyl betaine as a main surfactant. The Eye Irritation Score of cocamidopropyl betaine was 1.17, and due to its high usage frequency, its risk attention index was significantly higher than that of other ingredients, reaching 54. This result suggests that the irritation of CAPB in actual use may be higher than commonly recognized. Tween^®^-28, ranked second in usage frequency (30.77%), mostly appeared in slightly irritating and non-irritating formulations, with a low comprehensive irritation score (0.67) and a low risk attention index, indicating that Tween^®^-28 contributes little to the irritation of formulations and is a surfactant suitable for low-irritation formulations. SLES and SLA had the third-highest usage frequency (20.51%), and their comprehensive irritation scores and risk attention indices were also among the top, indicating that they contribute significantly to the irritation of formulations. In addition, PEG-120 methyl glucose dioleate had the highest comprehensive eye irritation score (1.33), but its low usage frequency (7.69%) meant that its risk attention index was not high. The statistical results suggest that CAPB, SLES, and SLA are commonly used ingredients in baby bath products and pose a high risk of irritation, warranting greater attention in formulations.

### 4.2. Analysis of Eye Irritation of Typical Ingredients in Baby Bath Products at Typical Usage Concentrations

The Eye Irritation Scores of the four categories of surfactants were calculated using Formula (2), yielding the following results: amphoteric surfactants, 0.86; anionic surfactants, 0.67; nonionic surfactants, 0.14; and cationic surfactants, 0 (Table 4).

Common literature indicates that anionic and cationic surfactants tend to be more irritating, whereas amphoteric and nonionic surfactants are generally milder [3]. In this study, nonionic surfactants had a comprehensive eye irritation score of only 0.14, indicating low overall irritation at the tested concentrations. Only decyl glucoside (5%) showed slight irritation, while the rest were non-irritating, which is consistent with the literature [31].

Among anionic surfactants, polyether sulfate salts exhibited stronger irritation, which is consistent with the data recorded by ECHA [32,33]. Amino acid-based anionic surfactants, carboxylates, and taurates showed higher overall mildness.

Surprisingly, the Eye Irritation Score of amphoteric surfactants is higher than that of anionic surfactants, indicating that under typical usage concentrations, the irritation of amphoteric surfactants may be higher than commonly recognized and should be considered when analyzing the sources of irritation in formulations. Among the nine amphoteric surfactants involved in this study, four exhibited mild irritation at the tested concentrations, namely CAPB (2%), SLA (5%), lauryl hydroxysultaine (2.4%), and lauramidopropyl hydroxysultaine (3.5%). Moreover, lauryl hydroxysultaine and lauramidopropyl hydroxysultaine from different sources all showed mild irritation. This data suggests that when considering how to reduce the irritation, more attention should be given to betaine-based amphoteric surfactants.

It is worth noting that the literature states that 5% sodium lauroyl sarcosinate causes no significant damage to the cornea [34]. However, the test results of the same concentration in this study showed it was mildly irritating, which is inconsistent with the literature. Additionally, different sources of the same surfactant (such as CAPB and SLA) at the same concentration showed a variety of results. The results indicate that the irritation of surfactant raw materials is not only related to structure, concentration, and pH, but may also be associated with production methods, quality control, and other factors.

Fragrances are mostly mixtures, and the experimental results showed that the four tested fragrances had different levels of irritation, with some showing mild irritation at low concentrations. Chinese regulations require that when fragrances are used in children’s cosmetics, potential allergenic fragrance components released by authoritative domestic and international institutions, such as Amyl cinnamal and Anisyl alcohol, should be labeled, but there are no specific requirements for the eye irritation of fragrances. Therefore, the incorporation of fragrances in baby bath products should be meticulously chosen and pre-tested to assess their potential for eye irritation.

### 4.3. Exploration of Reducing SLES Eye Irritation Through Combination

Although SLES has certain irritation potential, it remains the most commonly used main surfactant in baby bath products due to its good cleaning performance, compatibility with various other ingredients, chemical stability, and insensitivity to water hardness.

To explore strategies for reducing the eye irritation of SLES, we selected five different types of active substances to combine with SLES and compared the changes in eye irritation using both animal and cell models. The five combinations tested in this study were the small molecule panthenol, the cationic surfactant PQ-51, the nonionic surfactant Tween^®^-28, the anionic surfactant DCG, and the macromolecule MG-60.

The irritation results of the five combinations in animal and cell models were different (Table 5). In the animal model test (Draize test), panthenol, MG-60, and Tween^®^-28 were able to reduce irritation, but only Tween^®^-28 showed an irritation-reducing effect in the cell model test. In addition, PQ-51 did not change the irritation level in either the animal or cell model tests, while sodium cocoyl glutamate increased irritation in both models.

There are differences in physiological structure and metabolic absorption between animal and cell models. Animal ocular tissues have complex structures and physiological functions, with certain metabolic transformation and absorption capabilities, which can change the distribution and concentration of combinations in the eyes, thus providing space and possibility for the recovery of irritation reactions. In contrast, cell experiments involve only a single type of cell, and the concentration and state of the combination in the extracellular fluid are relatively stable, with direct action and short experimental time. Once the cell barrier is quickly destroyed, long-term reaction changes cannot be observed.

Studies have shown that the combination of nonionic surfactants with anionic surfactants can reduce the CMC of anionic surfactants, thereby reducing irritation [35]. We speculate that Tween^®^-28, as a nonionic surfactant, also combines with SLES to reduce the CMC value, making it easier for SLES to form micelles at the test concentration. This directly changes the concentration of free monomeric surfactants, thus showing a reduced irritation effect in both the animal model and the corneal epithelial cell model.

The compound MG-60 consists of maltotriose glucoside and hydrogenated starch hydrolysate, which possess multiple hydroxyl groups and exhibit strong hydrophilicity. Starch is known for its ability to reduce the release of pro-inflammatory factors (such as IL-6 and TNF-α) [36]. We speculated that MG-60 mitigated the inflammatory effects of SLES on ocular tissues in animal models, thus reducing the ocular irritation score. However, in the SIRC cell model, MG-60 may not have had sufficient time to exert its anti-inflammatory effects, as SLES had already caused direct damage to the cell membrane, leading to no significant change in SIRC cell viability. A similar combination is panthenol. According to the literature, panthenol can protect against oxidative stress damage, reduce inflammatory responses, and improve irritation symptoms [37]. However, at the SIRC cell level, the action of SLES is to directly destroy the cell membrane structure, causing cell lysis and inactivation, so panthenol did not show an irritation-reducing effect at the cellular level.

DCG is non-irritating to rabbit eyes when used alone at concentrations of 3–8%, but when combined with SLES, irritation increased in both the rabbit eye test and cell experiments. It is speculated that since both are anionic surfactants, the combination may interfere with the normal micelle formation process due to charge aggregation, resulting in unstable micelles or abnormal micelle aggregates, leading to an increase in the concentration of free surfactant monomers in the solution, and, thus, stronger irritation than when used alone.

PQ-51 is a bioaffinity material designed to mimic the cell membrane, capable of inhibiting the irritation of various surfactants to living organisms and cells, while also having super-hydration properties. In the combination tests of this study, it did not change the irritation of the main surfactant, possibly because the test concentration of PQ-51 was low, and it had little effect on the main surfactant, whose concentration was nearly three orders of magnitude higher.

## 5. Conclusions

This study performed Draize rabbit eye tests on 39 baby bath products from 2021 to 2022. Only 12 (30.77%) products were non-irritating, while 8 (20.51%) posed significant irritation risks. This highlights the need for better eye irritation safety in these products.

An innovative evaluation method using “Risk Attention Index” and “Eye Irritation Score” was applied to analyze ingredients in formulations. Results showed that SLES, CAPB, and SLA had high irritation scores and usage frequencies, requiring special attention.

Test raw materials were prepared at typical concentrations and pH values. Amphoteric surfactants had higher Eye Irritation Scores than anionic surfactants, with polyether sulfate and betaine being particularly irritating. Combination experiments revealed that SLES irritation decreased with panthenol, MG-60, and Tween^®^-28, but increased with DCG. Some fragrance components were also identified as potential irritants.

Inconsistent irritation levels were observed for the same ingredients from different manufacturers. This suggests that by-products and impurities generated during production, filling, storage, and transportation are also potential risk factors.

While employing the Draize test to obtain reliable in vivo data, this study acknowledges its ethical and translational limitations. Under the “3R” principles, in vitro methods represent an irreversible trend. Consequently, our Draize test data on commercial formulations provides value beyond singular results—serving as a critical benchmark for validating in vitro methods. As cosmetic formulations are complex mixtures, this high-quality in vivo dataset offers an essential reference for assessing alternative methods’ accuracy. Thus, our work not only determines irritation levels but also establishes a foundation for developing human-relevant, animal-free safety strategies, accelerating the transition to alternative testing.

Future work will focus on collecting more parameters from real-world usage, optimizing test sample configurations, and improving data utilization to conserve resources and reduce animal use. Further comparisons of surfactants from multiple sources are needed, with a focus on raw material quality control and production standards.

## Figures and Tables

**Table 1:** Eye irritation analysis of top 10 surfactants used in 39 commercial baby bath product formulations.

INCI Name	Total Number of Usage	Usage Rate ^1^	Number of Usage			Eye Irritation Score ^2^	Risk Attention Index ^3^
			Mildly Irritating	Slightly Irritating	Non-Irritating		
Cocamidopropyl Betaine (CAPB)	18	46.15%	4	13	1	1.17	54
PEG-80 Sorbitan Laurate (Tween^®^-28)	12	30.77%	1	6	5	0.67	21
Sodium Laureth Sulfate (SLES)	8	20.51%	3	4	1	1.25	26
Sodium Lauroamphoacetate (SLA)	8	20.51%	2	5	1	1.13	23
Decyl Glucoside	7	17.95%	0	6	1	0.86	15
TEA-Cocoyl Glutamate	5	12.82%	0	2	3	0.40	5
Disodium Cocoamphodiacetate	4	10.26%	2	0	2	1.00	10
Lauryl Hydroxysultaine	4	10.26%	0	2	2	0.50	5
PEG-7 Glyceryl Cocoate	4	10.26%	0	3	1	0.75	8
PEG-120 Methyl Glucose Dioleate	3	7.69%	1	2	0	1.33	10

Notes: ^1^ Usage Rate = Total number of usage/39. ^2^ Eye Irritation Score = [2 × Number of usage (Mildly irritating) + 1 × Number of usage (Slightly irritating) + 0 × Number of usage (non-irritating)]/Total number of usage. ^3^ Risk Attention Index = Eye Irritation Score × Usage Rate × 100.

**Table 2:** Draize test results database of ingredients used in baby bath products at the actual usage concentrations in the formulas.

Test Number	Test Chemical Name	Commercial Source	Classification	Test Concentration	pH	Draize Test Results
1	Decyl Glucoside	Company 1	Nonionic surfactant	5.00%	5.5–6.0	Slightly irritating
2	Coco-Glucoside (And) Glyceryl Oleate	Company 1	Nonionic surfactant	3.00%	5.5–6.0	Non-irritating
3	Lauryl Glucoside	Company 1	Nonionic surfactant	3.00%	5.5–6.0	Non-irritating
4	Capryl/Capramidopropyl Betaine	Company 2	Amphoteric surfactant	2.00%	5.5–6.0	Non-irritating
5			Amphoteric surfactant	5.00%	5.5–6.0	Slightly irritating
6	Cocamidopropyl Hydroxysultaine	Company 3	Amphoteric surfactant	1.80%	5.5–6.0	Non-irritating
7	Lauryl Hydroxysultaine	Company 4	Amphoteric surfactant	2.40%	5.5–6.0	Mildly irritating
8		Company 5	Amphoteric surfactant	2.40%	5.5–6.0	Mildly irritating
9	Cocamide Methyl MEA	Company 5	Amphoteric surfactant	2.00%	5.5–6.0	Non-irritating
10	Sodium PEG-7 Olive Oil Carboxylate	Company 6	Anionic surfactant	1.44%	5.5–6.0	Non-irritating
11	Sodium Trideceth Sulfate	Company 7	Anionic surfactant	3.25%	5.5–6.0	Mildly irritating
12	TAE-Cocoyl Glutamate	Company 8	Anionic surfactant	5.00%	5.5–6.0	Non-irritating
13	Sodium Taurine Cocoyl Methyltaurate	Company 9	Anionic surfactant	3.00%	5.5–6.0	Non-irritating
14			Anionic surfactant	5.00%	5.5–6.0	Slightly irritating
15	Laureth-5 Carboxylic Acid	Company 10	Anionic surfactant	3.00%	5.5–6.0	Non-irritating
16			Anionic surfactant	5.00%	5.5–6.0	Slightly irritating
17	Ammonium Laureth Sulfate	Company 5	Anionic surfactant	5.00%	5.5–6.0	Mildly irritating
18	Sodium Lauroyl Sarcosinate	Company 4	Anionic surfactant	5.00%	5.5–6.0	Mildly irritating
19	PPG-5-Ceteth-20	Company 11	Nonionic surfactant	5.00%	6.0–6.5	Non-irritating
20	Polyglyceryl-10 Isostearate	Company 12	Nonionic surfactant	5.00%	6.0–6.5	Non-irritating
21	Polyglyceryl-10 Laurate	Company 13	Nonionic surfactant	3.00%	6.0–6.5	Non-irritating
22	PEG-120 Methyl Glucose Dioleate	Company 14	Nonionic surfactant	1.00%	6.0–6.5	Non-irritating
23	Cocamidopropyl Betaine	Company 15	Amphoteric surfactant	2.00%	6.0–6.5	Mildly irritating
24		Company 1	Amphoteric surfactant	2.00%	6.0–6.5	Slightly irritating
25		Company 16	Amphoteric surfactant	2.00%	6.0–6.5	Slightly irritating
26		Company 2	Amphoteric surfactant	2.00%	6.0–6.5	Non-irritating
27	Sodium Cocoamphoacetate	Company 2	Amphoteric surfactant	1.90%	6.0–6.5	Non-irritating
28			Amphoteric surfactant	3.20%	6.0–6.5	Slightly irritating
29	Sodium Lauroamphoacetate	Company 3	Amphoteric surfactant	1.50%	6.0–6.5	Non-irritating
30			Amphoteric surfactant	3.20%	6.0–6.5	Non-irritating
31		Company 15	Amphoteric surfactant	5.00%	6.0–6.5	Mildly irritating
32		Company 1	Amphoteric surfactant	5.00%	6.0–6.5	Slightly irritating
33	Disodium Cocoamphodiacetate	Company 17	Amphoteric surfactant	1.90%	6.0–6.5	Non-irritating
34		Company 2	Amphoteric surfactant	3.20%	6.0–6.5	Non-irritating
35			Amphoteric surfactant	5.00%	6.0–6.5	Non-irritating
36	Polyquaternium-10	Company 18	Cationic surfactant	0.30%	6.0–6.5	Non-irritating
37	Sodium Methyl Cocoyl Taurate	Company 19	Anionic surfactant	1.50%	6.0–6.5	Non-irritating
38			Anionic surfactant	3.00%	6.0–6.5	Non-irritating
39	Sodium Cocoyl Alaninate	Company 8	Anionic surfactant	1.50%	6.0–6.5	Slightly irritating
40	Disodium Cocoyl Glutamate	Company 4	Anionic surfactant	4.50%	6.0–6.5	Non-irritating
41			Anionic surfactant	8.00%	6.0–6.5	Non-irritating
42	Sodium Laureth Sulfate	Company 5	Anionic surfactant	5.00%	6.0–6.5	Slightly irritating
43	Lauramidopropyl Hydroxysultaine	Company 4	Amphoteric surfactant	3.50%	6.0–7.0	Mildly irritating
44		Company 15	Amphoteric surfactant	3.50%	6.0–7.0	Mildly irritating
45		Company 3	Amphoteric surfactant	3.50%	6.0–7.0	Mildly irritating
46	Sodium Lauroyl Glutamate	Company 4	Anionic surfactant	5.00%	6.5–7.0	Slightly irritating
47	Potassium Cocoyl Glycinate	Company 20	Anionic surfactant	4.50%	7.0–8.0	Non-irritating
48	Sodium Cocoyl Glycinate	Company 17	Anionic surfactant	4.50%	7.0–8.0	Slightly irritating
49	Phenoxyethanol	Company 1	Preservative	0.50%	No adjustment	Non-irritating
50			Preservative	1.00%	No adjustment	Non-irritating
51			Preservative	1.00% (Do not rinse off)	No adjustment	Non-irritating
52	Hydroxyacetophenone	Company 21	Preservative	0.50%	No adjustment	Non-irritating
53	Caprylhydroxamic Acid	Company 22	Preservative	1.50%	No adjustment	Non-irritating
54	Orange Blossom Fragrance	Company 23	Fragrance	0.10%	No adjustment	Slightly irritating
55	Red Mandarin Essential Oil	Company 24	Fragrance	0.10%	No adjustment	Non-irritating
56	Fragrance 369	Company 25	Fragrance	0.20%	No adjustment	Mildly irritating
57	Fragrance 440095	Company 21	Fragrance	0.20%	No adjustment	Non-irritating
58	Dipropylene Glycol	Company 26	Humectant	0.02%	No adjustment	Non-irritating

**Table 3:** The Draize test results of 5% SLES compounded with different active ingredients.

Main Surfactant	Compounded Active Ingredients	Concentration	pH	Draize Test Result
5% SLES	/	/	6.0–6.5	Slightly irritating
	Panthenol	1%	6.0–7.0	Non-irritating
	Polyquaternium-51 (PQ-51)	0.007%	6.0–7.0	Slightly irritating
	Maltooligosyl glucoside/hydrogenated starch hydrolysate (MG-60)	1.48%	6.0–7.0	Non-irritating
	Tween^®^-28	3.5%	5.5–6.0	Non-irritating
	Disodium cocoyl glutamate (DCG)	3%	5.5–6.0	Mildly irritating

**Table 4:** Eye irritation analysis of common surfactants used in baby bath products at typical usage concentrations and pH levels.

Classification	Total Number of Tests	Number of Eye Irritation Results			Eye Irritation Score ^1^
		Mildly Irritating	Slightly Irritating	Non-Irritating	
Anionic surfactant	18	3	6	9	0.67
Amphoteric surfactant	22	7	5	10	0.86
Nonionic surfactant	7	0	1	6	0.14
Cationic surfactant	1	0	0	1	0

Note: ^1^ Eye Irritation Score = [2 × Number of use (Mildly irritating) + 1 × Number of use (Slightly irritating) + 0 × Number of use (non-irritating)]/Total Number of use.

**Table 5:** Comparison of results of SLES compounded with different active ingredients in animal and cellular models.

Main Surfactant	Compounded Active Ingredients	Concentration in Animal Model	Change in Irritation Grade Compared to Before Compounding (Based on Animal Model)	Concentration in Cellular Model	Change in Irritation Grade Compared to Before Compounding (Based on SIRC Model)
5% SLES (in animal model) 0.01% SLES (in SIRC model)	PQ-51	0.007%	Not change	0.00007%	Not change
	Panthenol	1%	Decreased	0.01%	Not change
	MG-60	1.48%	Decreased	0.0148%	Not change
	Tween^®^-28	3.50%	Decreased	0.035%	Decreased
	DCG	3%	Increased	0.03%	Increased

## Data Availability

The data that support the findings of this study are available from the corresponding authors [G.H. and J.T.], upon reasonable request.

## Supplementary Materials

The following supporting information can be downloaded at https://www.mdpi.com/article/10.3390/toxics13110948/s1. The details for Animal Selection and Housing, Draize Test Experimental Procedure, and Inclusion and Exclusion Criteria, and Table S1 (Grading of acute eye irritation/corrosion test results) are shown in the Supplementary Materials.

## Abbreviations

The following abbreviations are used in this manuscript:

SIRC cells	Rabbit corneal epithelial cells
HET-CAM	Hen’s egg test-chorioallantoic membrane
BCOP	Bovine corneal opacity and permeability
IATA	Integrated assessment testing approach
ECETOC	European Centre for Toxicology and Ecotoxicology of Chemicals
DRD	Draize Eye Test Reference Database
REACH	Registration, Evaluation, Authorisation and Restriction of Chemicals
ECHA	European Chemicals Agency
TNF-α	Tumor necrosis factor-α
MEM	Minimum essential medium
STE	Short-time exposure
OD450	Absorbance at 450 nm
GFP	Green fluorescent protein
CAPB	Cocamidopropyl betaine
SLES	Sodium laureth sulfate
SLA	Sodium Lauroamphoacetate
Tween^®^-28	PEG-80 sorbitan laurate
DCG	Disodium cocoyl glutamate
MG-60	Maltooligosyl glucoside/hydrogenated starch hydrolysate
