# Supplementary material for: From Safety Evaluation to Influencing Factors Analysis: A Comprehensive Investigation on Ocular Irritation of Baby Bath Products
Source: Toxics. 2025 Nov 3;13(11):948. doi: 10.3390/toxics13110948 (PMC12656482; doi:10.3390/toxics13110948)
Supplement: Supplementary file 1 [file toxics-13-00948-s001.zip › toxics-3945744-supplementary.pdf]

Supplementary Materials

Qidi He[1,2,,†], Yurong Zhong[3,†], Peining Li[1,2,] Yanhua Guo[1,2], Chengkai Mei[1,2], Dongmei Xu[3], Erping Yan[3], Shaofeng Xi[1,2], Guoshan He[1,2*], Jianhua Tan[1,2*]

[1] Guangzhou Quality Testing and Inspection Institute, National Quality Supervision and Testing Center for Cosmetics (Guangzhou), 510000, Guangzhou, China; anchorgreen@foxmail.com (Q.H.); lpn1117@163.com (P.L.); guoyanhuasysu@foxmail.com (Y.G.); vickiemay@163.com (C.M.); 13922262758@163.com (S.X.)

[2] Collaborative Innovation Center for NQI-Quality Safety of Guangzhou, 510000, Guangzhou, China

[3] Kangaroo Mommy Research Centre, Guangdong Kangaroo Mommy Biotechnology Co., Ltd., 510000, Guangzhou, China; yulingqingheng@163.com (Y.Z.); v1126942960@163.com (D.X.); jsyan6944@icloud.com (E.Y.)

* Correspondence: hsame@sina.com.cn (G.H.), tanjianhua0734@aliyun.com (J.T.)

† These authors contributed equally to this work.

S1.Materials and Methods

S1.1 Animal Selection and Housing

The New Zealand rabbits were all purchased from the Guangdong Provincial Medical Experimental Animal Center (Sanshui Base, China). They were in good health, lively, and responsive, with normal reactions to external stimuli. They did not show signs of lethargy, restlessness, or depression. Their fur was smooth, and they were well-developed. All were female, with a body weight of 2.0kg to 2.2kg.

After purchase, the animals were housed individually in the conventional environment animal room of our institute [Experimental Animal Use License No. SYXK (Yue) 2018-0137]. The temperature was maintained at 18℃ to 26℃, and the relative humidity was kept at 30% to 70%. The feed was provided by Beijing Keao Xieli Feed Co.,Ltd. (Beijing, China) [Production License No.: SCXK (Jin) 2020-0004].

Members of the research team and the veterinarian conducted two clinical inspections of the animals daily. The health status of the animals was monitored through routine assessments of their condition, food intake, and water consumption.

In this study, three conventional-grade New Zealand rabbits (female) were selected for the acute eye irritation test of each test substance. A total of 39 products, 58 single-component samples, and 5 compound samples were tested, using 306 rabbits in total. According to the position of the rabbit cage on the rack, each cage was assigned a numerical code. Each rack had 4 columns of rabbit cages, with 3 cages per column. Each column formed a group, with 3 rabbits per group.

The animal experiments were approved by the Animal Welfare and Ethics Committee of Guangzhou Quality Supervision and Inspection Institute. The animal experiment ethics review approval numbers are IACUC2021-12-01 and IACUC2022-12-02.

S1.2 Draize Test Experimental Procedure

The eye irritation evaluation of the products and raw materials was conducted using the Draize test method modified based on OECD Guideline 405. New Zealand rabbits were randomly assigned to groups of three after passing quarantine inspection for the evaluation of eye irritation of the test substances. A volume of 0.1 mL of the test substance was instilled into the left conjunctival sac of each of the three rabbits. The upper and lower eyelids were passively closed for 1 second, and the right eye was left unwashed as a control.

For each animal, two different researchers were involved. The first researcher performed the random sampling and scoring operations and was the only person who knew the experimental treatment group allocation. The second researcher was responsible for scoring.

The eyes of the animals were examined for cornea, iris, or conjunctiva at 1, 24, 48, 72 hours, and on days 4 and 7 after instillation of the test substance. The assessment of rabbit eye damage includes the conjunctiva (congestion, swelling), the cornea (degree of opacity), and the iris (structural changes, reaction to light).

The average scores of the conjunctiva, cornea, and iris for each group of three rabbits were calculated, and the irritation was determined according to the criteria in Table S1.

Table S1. Grading of acute eye irritation/corrosion test results

Eye irritation results	Grades	Criteria
Non-irritation	Non-irritating	The integral value of the cornea, iris, and conjunctiva of the animal is 0.
Reversible irritation	Slightly irritating	The reaction integral value is between non-irritating and mildly irritating.
	Mildly irritating	The average irritation reaction integral value in 2/3 of the animals: corneal opacity ≥ 1; iris ≥ 1; conjunctival hyperemia ≥ 2; conjunctival edema ≥ 2, and the above irritation reaction integral value is completely restored within ≤ 7 days.
	Irritating	The average irritation reaction integral value in 2/3 of the animals: corneal opacity ≥ 1; iris ≥ 1; conjunctival hyperemia ≥ 2; conjunctival edema ≥ 2, and the above irritation reaction integral value is completely restored within < 21 days.
Irreversible damage	Corrosive	①The integral value of the cornea, iris, and/or conjunctiva of the animal is greater than 0 on day 21.<br>②The average eye irritation reaction integral value in 2/3 of the animals: corneal opacity ≥ 3 and/or iris = 1.5.

S1.3 Inclusion and Exclusion Criteria

Animals that did not show severe reactions in the conjunctiva, cornea, or iris during the experiment were included in the study. Animals that showed severe depression or distress at any stage of the experiment should be euthanized and excluded. Animals with corneal perforation, corneal ulcer, corneal score of 4 for more than 48 hours, absence of light reflex for

more than 72 hours, conjunctival ulcer, gangrene, or necrosis should be euthanized and excluded.

All test substances in this study showed no severe reactions in the rabbit eye irritation tests, and there were no cases of excluding experimental animals.
